# Erythematous Linear Lesion on the Course of Superficial Fibular Nerve After the Topical Application of Black Henna: A Case Report

**DOI:** 10.7759/cureus.36697

**Published:** 2023-03-26

**Authors:** Ahmed F Alkandari, Abrar A Alawadhi, Fatma A Alawadhi, Alyaa Mousa, Sampath Madhyastha

**Affiliations:** 1 Department of Anatomy, Kuwait University, Jabriya, KWT; 2 Department of Surgery, Al-Adan Hospital, Al-Ahmadi, KWT

**Keywords:** cutaneous neuritis, superficial fibular nerve, ppd, para phenylenediamine, black henna

## Abstract

Henna is commonly used in body arts, where it produces orange-brown color. It is often mixed with chemicals such as para-phenylenediamine (PPD) to fasten the dyeing process and produce a black color. However, PPD has many allergic and toxic effects. We present a case of henna-induced cutaneous neuritis, which is not reported before. A 27-year-old female presented to our hospital, complaining of pain in her left great toe after applying black henna. Upon examination, the proximal nail fold was inflamed, and an erythematous non-palpable tender lesion was noticed on the dorsum of the foot. The lesion had an inverted-Y shape that was confined to the course of the superficial fibular nerve. Cutaneous nerve inflammation was favored after excluding all the anatomical structures in the region. Black henna should be avoided since it contains PPD, which can be absorbed through the skin and affect the underlying cutaneous nerves.

## Introduction

The application of topical cosmetics over the skin as temporary body art is common in many parts of the world, including the Arabian Gulf [1]. Henna or Hina, a dried leaf of the Lawsonia Inermis plant (family Lythraceae), is used to dye the skin of palms, dorsum of the hand, soles, dorsum of the feet, hair, and nails [2]. Henna has been used to beautify women's bodies during wedding ceremonies and other social celebrations in Arab countries and India. However, some individuals may develop allergic reactions to henna, which is safer than black henna. Adding chemicals to henna makes it toxic and fatal [3]. Paraphenylenediamine (PPD) is the commonly used ingredient in most hair dye preparations, including commercially available henna, where its concentration may reach up to 30% [4], despite the regulations to restrict it to 6% [5]. Furthermore, the level of PPD in black henna used for tattooing is higher than that found in hair dyes [6]. The terms 'Kala Pathar' and 'paraphenylenediamine' are interchangeably used in literature. Kala Pathar, meaning 'black rock' in Nepali, is a mountain in the Nepalese Himalayas [7], from which the PPD is extracted as its most active ingredient [6]. PPD is an aromatic amine derived from para-nitroaniline that belongs to oxidizing dyes [8]. The pure form of PPD is white to yellow in color, which darkens on exposure to air via oxidation [6]. The easy availability and low cost of PPD-containing henna make it a widely used substance and a preferred agent for suicidal and homicidal intents [9, 10]. In Kuwait, PPD-containing henna is available in two forms; a powder and a paste. While the paste form of henna can be applied directly on the skin, the powder form is mixed with water before its application. PPD is mainly used as an element of oxidative hair coloring products and temporary tattoos to fasten the dyeing process and produce a black color [11-13]. It can penetrate the hair shaft and undergo oxidative reactions, causing hair discoloration [14]. PPD is also known to be used in tissue preparation for histological studies on nerves [15]. It can enhance osmium staining of myelin sheaths of peripheral nerves by chelating the osmium in tissues fixed with osmium tetroxide [16]. The systemic and local side effects of oral ingestion of PPD-containing hair dyes are explained in the Discussion section. Motor neuropathy after ingesting black henna is rarely reported [17]. Topical cosmetics penetrate the intact skin by trans-epidermal or trans-appendageal route [18, 19]. The damaged skin can allow substances to penetrate easily [20], as per acute eponychia. Acute eponychia, an inflammation of the proximal nail fold, is caused by a minor trauma that disrupts the protective barrier between the nail fold and plate, leaving it vulnerable to bacterial invasion and infection [21]. Reports of allergic contact dermatitis to natural henna are very rare in the literature, and often assumed that natural henna is a very weak skin allergen [22]. We, therefore, present a case of cutaneous nerve inflammation after the topical application of PPD-containing henna facilitated by a defect in the skin barrier. This study was previously presented as a poster and published as a meeting abstract at the 25th Health Sciences Center (HSC) Conference during March 16-18, 2021.

## Case presentation

**Figure 1:**
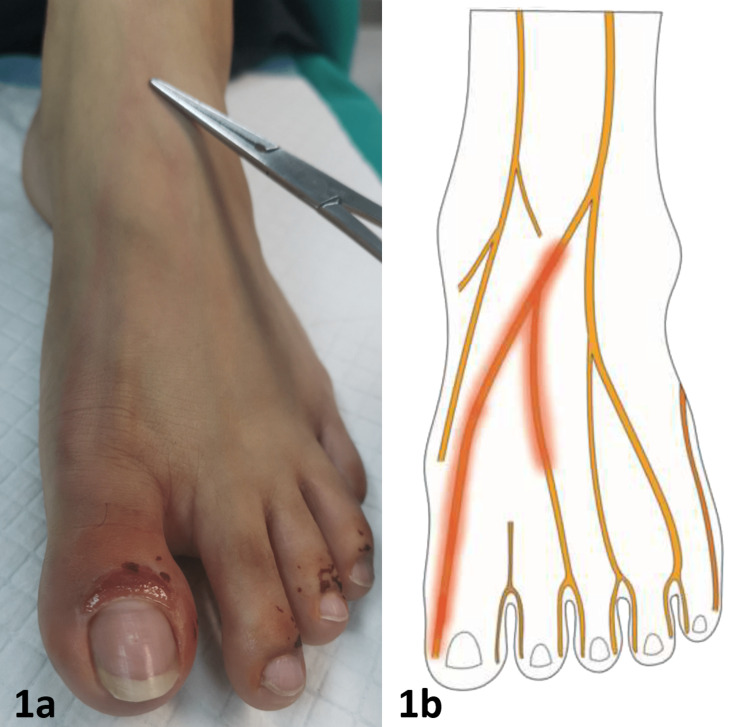
Clinical and anatomical correlation. (a) Dorsum of the patient's left foot: showing the inverted 'Y' lesion running along the course of the superficial fibular nerve. (b) Sensory innervation of the dorsum of the foot showing the affected branches of the nerve (Courtesy of Sampath Madhyastha, the corresponding author).

A 27-year-old woman with no past medical history presented to the casualty operation theater at Al-Adan hospital, complaining of pain in her left great toe for the past day. History taking revealed no recent trauma to the affected foot. Upon examination, the proximal nail fold of the left great toe was inflamed (eponychia). The nail plate showed normal morphology, but the proximal nail folds of the toes had blackish macules, which upon asking, they were remnants of black henna applied about 15 days before. Coincidentally, an erythematous non-palpable tender lesion was found on the dorsum of the affected foot. The lesion had the shape of an inverted 'Y,' which ran along the course of the superficial fibular peroneal nerve, as shown in Figure 1.

The patient denied any history of trauma to the nail. The patient also denied any history of allergy in the form of atopic dermatitis. A general physical examination showed no fever or regional lymphadenopathy. Motor examination showed no muscle weakness or pain upon plantar flexion. Sensory examination showed no paresthesia, hyperesthesia, or burning sensation. However, the patient experienced an electrical touch while pinching the skin over the course of the affected nerve. The eponychia was deroofed, the fluid was drained, and the patient was discharged with oral antibiotics and painkillers.

## Discussion

The systemic and local side effects of oral ingestion of PPD-containing hair dyes are listed in Table 1.

**Table 1:** Systemic and local side effects of ingestion of PPD-containing hair dye. PPD: Paraphenylenediamine.

Onset of symptoms	
Early symptoms (due to hypersensitivity and anaphylactic reaction)	Purple discoloration of teeth or gums. Contact dermatitis and blepharoconjunctivitis. Epigastric pain and vomiting. Angioneurotic edema and respiratory distress. Muscular cramps and convulsions. Anaphylactic shock.
Late symptoms (due to toxic effects on various body systems)	Heart: acute myocarditis, myocardial infarction, and cardiac arrest. Muscles: skeletal muscle fatigue, limb rigidity, and rhabdomyolysis. Kidneys: acute tubular necrosis and acute renal failure. Liver: acute hepatitis and acute hepatic failure. Central nervous system: drowsiness, lethargy, seizure, coma, critical illness neuropathy, pure motor paralysis, syndrome of hypertonia and hyperreflexia, and optic neuritis.

To the authors' best knowledge, this is the first documented case of cutaneous nerve inflammation after the topical application of henna in humans. Black henna contains PPD [6], which can be absorbed through the skin [23]. Damaged skin, in turn, can facilitate the absorption of topical cosmetics [20]. In our case, the erythematous Y-shaped lesion observed along the course of the superficial fibular nerve was induced by PPD found in black henna, which was applied 15 days ago. The defect in the skin barrier, in terms of eponychia, facilitated the absorption and diffusion of PPD into the skin and underlying nervous tissue. The mechanism by which PPD affects the nervous tissue could be due to two presumptions: neuro-receptor damage or myelin sheath damage. In neuro-receptor damage, the hypothesis is based on the idea that the 'non-encapsulated' free nerve endings are vulnerable to mechanical and chemical damage from the environment [24]. While in myelin sheath damage, it is based on the affinity of PPD to affect and stain phospholipids as it does in the histological preparations for nervous tissues [15]. Waggas A and Arabia S [1], in their animal study, found that the topical application of PPD (2 mg/kg) on the fur of brown rats for five weeks can cause a significant decrease in the content of monoamines in all brain regions, causing various CNS symptoms such as ataxia and seizures. Infectious or mechanical tenosynovitis of the extensor tendons, thromboangiitis obliterans, and superficial lymphangitis was ruled out in this patient. Kano Y et al. [25] reported a linear lesion caused by superficial lymphangitis after the onset of paronychia in the thumb, possibly due to bacterial infection. However, in our case, the lesion was not palpable and did not cause regional lymph node enlargement, which excludes superficial lymphangitis. Nevertheless, a final diagnosis could not be made with certainty since a skin biopsy was not done. Cutaneous nerve inflammation was favored after excluding the other anatomical structures in the region and because of the electrical touch experienced by the patient while pinching the skin over the course of the affected nerve. The limitation of this report includes a culture and sensitivity test for pus would confirm the diagnosis of eponychia. Also, a patch skin test for PPD could exclude contact allergy. Skin biopsy for the lesion could confirm the diagnosis of cutaneous neuritis.

## Conclusions

Using black henna should be avoided since it contains PPD, which can be absorbed through the skin and affect the underlying nervous tissue. Further studies will be useful to consider PPD as a neuro-inflammatory agent in producing peripheral nerve inflammation while designing research study designs.
